# Studies on the Antifatigue Activities of *Cordyceps militaris* Fruit Body Extract in Mouse Model

**DOI:** 10.1155/2015/174616

**Published:** 2015-08-17

**Authors:** Jingjing Song, Yingwu Wang, Meiyu Teng, Guangsheng Cai, Hongkai Xu, Hanxiao Guo, Yang Liu, Di Wang, Lesheng Teng

**Affiliations:** College of Life Sciences, Jilin University, Changchun 130012, China

## Abstract

*Cordyceps militaris* has been used extensively as a crude drug and a folk tonic food in East Asia due to its various pharmacological activities. Our study aims to investigate the effect of *Cordyceps militaris* fruit body extract (CM) on antifatigue in mouse model. Two week CM administration significantly delayed fatigue phenomenon which is confirmed via rotating rod test, forced swimming test and forced running test. Compared to nontreated mouse, CM administration increased ATP levels and antioxidative enzymes activity and reduced the levels of lactic acid, lactic dehydrogenase, malondialdehyde, and reactive oxygen species. Further data suggests that CM-induced fatigue recovery is mainly through activating 5′-AMP-activated protein kinase (AMPK) and protein kinase B (AKT)/mammalian target of rapamycin (mTOR) pathways and regulating serum hormone level. Moreover, CM-enhanced the phosphorylation of AMPK contributes to its antioxidant effect. Our data provides experimental evidence in supporting clinical use of CM as an effective agent against fatigue.

## 1. Introduction

Fatigue is defined as a failure to maintain the required or expected force or power output [1], which has been classified into mental and physical fatigue [2]. Physical fatigue is commonly associated with the elevated stress level caused by a modern life style and also related to exercise-reduced maximal force-generating capacity of muscle [3]. As reported, energy metabolism and reactive free radicals accumulation are involved in the pathophysiology of fatigue [4, 5]. When fatigue happens, it will gradually accumulate and eventually lead to overstrain, endocrine disorders, a weakened immune system, even organ lesions [6].

5′-AMP-activated protein kinase (AMPK), a key regulator for body energy balance, acts to suppress anabolic ATP-consuming pathways [7]. Moreover, AMPK activation counteracts oxidative stress via inhibiting NAD(P)H oxidase-derived reactive oxygen species (ROS) accumulation. On the other hand, excessive exercise leads to the production of endogenous oxidative free radicals and lipid peroxidation, which can damage the cell membrane system [8–10]. The increment of mitochondrial lipid peroxidation products, such as malondialdehyde (MDA), covalently bonds to the mitochondria membrane, which leads to the accumulation of intracellular calcium ion and the reduction of ATP [11]. Enzymatic and nonenzymatic antioxidants play a vital role in protecting tissues from excessive oxidative damage during exercise [10]. The enhanced activity of antioxidant enzyme prolongs exercise performance and reduces physical fatigue [12]. Additional oral doses of antioxidants prevent or reduce oxidative stress, decrease muscle damage, and improve exercise performance [8, 13]. Searching natural antioxidants originated from plants against fatigue has been a hot topic.

As fatigue is becoming serious in modern society and pharmacological drugs or therapies cannot satisfy the need of people, potential alternatives from herbs have been increasing worldwide.* Rhodiola rosea*, a commonly used antihypoxia traditional Chinese medicine, also displays the enhancement of physical ability [13].* Cordyceps militaris*, one of the most important traditional Chinese medicines, has been used extensively as a crude drug and a folk tonic food in East Asia [14]. Due to its kind of bioactive substance including cordycepin, polysaccharides, ergosterol, and mannitol [15],* Cordyceps militaris* possesses anti-inflammatory, antioxidant, antiaging, and antitumor effects [14]. Our previous study successfully demonstrated that* Cordyceps militaris* mycelium obtained from submerged fermentation displays excellent antidiabetic and antinephropathic activities [16]. Purified* Cordyceps militaris* polysaccharides show the similar antihypoxia effect as rhodiola oral liquid [17]. However, few studies report the antifatigue effect of* Cordyceps militaris*.

Based on previous research, we hypothesized that* Cordyceps militaris* fruit body polysaccharides-enriched extract may display the antifatigue effect. To test this hypothesis, we investigated the antifatigue and antioxidant activities of* Cordyceps militaris* via mouse model. During the whole experiment, the concentration of ATP and the activities of oxidant related enzymes in serum, muscle, and liver were detected. The hormones in both female and male mouse were further determined. To analyze its underlying mechanism, relevant signaling including protein kinase B (AKT), extracellular signal-regulated kinase (ERKs), mammalian target of rapamycin (mTOR) and AMPK in liver were measured.

## 2. Materials and Methods

### 2.1. *Cordyceps militaris* Extract Preparation

*Cordyceps militaris* fruit body was purchased from Qianxiang Co., Ltd. (Shenyang, China). The aqueous extract from* Cordyceps militaris* fruit body was extracted with 10 volumes of distilled water at 45°C for 3 h firstly. After centrifugation, the residue was extracted at 80°C for another 3.5 h. Merging the two extracts, the supernatant was sequentially concentrated in an evaporator under reduced pressure and further freeze-dried to produce the solid aqueous extract (CM). Our preliminary experiments revealed that polysaccharides-enriched* Cordyceps militaris* fruit body water extract contains 29.1% polysaccharides, 20.5% total proteins, 6.1% cordycepic acid, 0.2% adenosine, and 0.4% cordycepin.

### 2.2. Animal Care

The experimental animal protocol used in the study was approved by the lab animal center of Jilin University (SCXK(JI)-2011-0003). The Institution Animal Ethics Committee reviewed the entire animal protocol prior to conducting the experiments. KunMing (KM) mice (6 weeks, 18–22 g, equal numbers of male and female, purchased from Norman Bethune University of Medical Science, Jilin University, Jilin, China) were maintained on a 12 h light/dark cycle (lights on 07:00–19:00) at 23 ± 1°C with water and food available* ad libitum*. Eight hours before the experiment, the animals were deprived for food with free access to water. All the experiments were performed in a quiet room, and each animal was used only once.

### 2.3. Measurement of Antifatigue Capacity

After a week adaptation, KM mice were divided into five groups randomly (*n* = 20/group; equal numbers of male and female) and orally treated with double distilled (D.D.) water (serving as control group), 0.5 g/kg* Rhodiola rosea* extract (Pro; serving as positive group and purchased from Tongrentang, Beijing, China), and CM at doses of 0.5 g/kg, 1.0 g/kg, and 2.0 g/kg once a day for 2 weeks. Doses and administration route were selected based on previous experiments performed in our laboratory. At the end of drug administration, following experiments were performed.

*Forced Running Test.* The endurance on a treadmill of all groups of mice was tested, which allowed them to run at a set speed of 15 rpm for 1 min. After 3-time practice, mice were putting on the treadmill with a set speed of 20 rpm. During the experiment, once the mouse stopped running, it would be shocked by the electrode. If the mouse was consecutively shocked 5 times, it is considered as running to exhaust. The total running time was recorded to evaluate their performance.

*Rotating Rod Test*. A fatigue turning device was used to test mouse performance after 14-day CM administration. Before formal test, 3-time exercise training at a speed on 15 rpm for 1 min was applied. During the fatigue analysis, mice were putting on the turning device at a speed of 20 rpm. The total duration on the rod was recorded.

*Weight-Loaded Forced Swimming Test.* After 14-day CM treatment, a weight-loaded forced swimming test was used to evaluate the endurance and performed of each mouse. Mice loaded with 15% of their body weight swam in water with 22 ± 1°C temperature and 30 cm depth. Exhaustion time was recorded from the beginning of swimming to the point at which mice failed to return to the water surface within 15 s.

### 2.4. Sample Collection

Overnight fasted KM mice (*n* = 24/group; equal numbers of male and female) were orally treated with D.D. water (serving as vehicle group), 0.5 g/kg* Rhodiola rosea* extract (serving as positive group), and CM at doses of 0.5 g/kg, 1.0 g/kg, and 2.0 g/kg once a day for 2 weeks. 60 min after last treatment, 12 mice (equal numbers of male and female) in each group were forced to swim in water with 22 ± 1°C temperature and 30 cm depth. After 10 min recess, blood samples were collected from caudal vein in all mice. At the end of the experiment, mice were sacrificed. Liver and muscle were quickly dissected, washed in ice-cold physiological saline solution, and homogenized in D.D. water.

### 2.5. Parameters Determination

The activities of superoxide dismutase (SOD), glutathione peroxidase (GSH-Px), and lactic dehydrogenase (LDH) and the levels of malondialdehyde (MDA) and lactic acid (LD) in serum, liver, and muscle and ATP and reactive oxygen species (ROS) in liver and muscle were determined according to the procedures provided by the related assay kits (NanJing Biotechnology Co. Ltd., NanJing, China). For tissue parameters determination, the tissues were homogenized in D.D. water via microcentrifuge tube homogenate (VWR America) and centrifuged at 902 ×g for 10 min. The protein concentration was detected via Bradford method.

### 2.6. Hormone Determination

After 14-day CM and* Rhodiola rosea* extract treatment, the levels of estradiol (E2), testosterone (T), and cortisol in serum were measured using enzyme-linked immunosorbent assay (ELISA) according to the procedures provided by the related assay kits (Calbiotech, USA).

### 2.7. Western Blot Analysis

Liver tissue samples were homogenized with 5–10 volumes of lysis buffer (Sigma-Aldrich, USA) containing 1 mM phenylmethanesulfonyl fluoride (PMSF) and 1X protease inhibitor cocktail (Sigma-Aldrich, USA). The homogenate was centrifuged at 10836 ×g for 10 min and supernatants were used as the whole protein extract. Total protein was estimated using Bradford method. About 30 *μ*g of the liver protein was separated by 10% SDS-PAGE and then transferred onto a nitrocellulose membrane (0.45 *μ*m, Bio Basic, Inc.) with miniprotein two-gel electrophoresis system (Bio-Rad, USA). The transferred membrane was blocked through incubation with 5% bull serum albumin (BSA) for 3 h at room temperature. The transferred membranes were then blotted with the following primary antibodies at 4°C overnight at dilution of 1 : 1000: phosphor-mTOR (P-mTOR), total-mTOR (T-mTOR), P-AKT, T-AKT, P-AMPK, T-AMPK, P-ERKs, T-ERKs, and glyceraldehyde-3-phosphate dehydrogenase (GAPDH) (Cell Signaling Technology, Beverly, MA), followed by treatment with horseradish peroxidase-conjugated secondary antibodies diluted 1 : 2000 (Santa Cruz, USA). Immunoreactive bands were visualized with an ECL detection system (GE Healthcare, UK). The intensity of the bands was quantified by scanning densitometry using software Quantity One-4.5.0.

### 2.8. Statistical Analysis

All data were expressed as mean ± SD. One-way analysis of variance (ANOVA) was used to detect statistical significance followed by post hoc multiple comparisons (Dunn's test) using SPSS 16.0 software (IBM corporation, Armonk, USA). A *P* value < 0.05 was considered to be statistically significant. Graphs were plotted with the OriginPro 8.5 software.

## 3. Results

### 3.1. The Antifatigue Capacity of* Cordyceps militaris*

**Figure 1:**
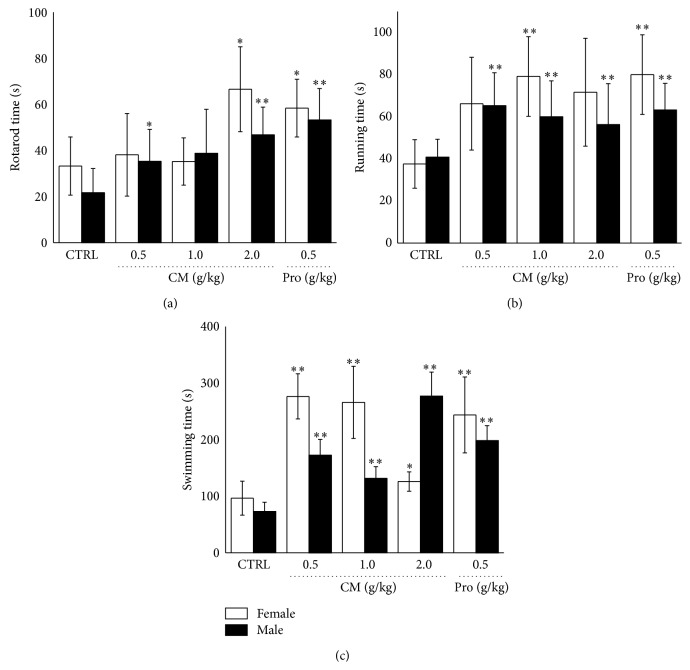
The antifatigue effects of* Cordyceps militaris* fruit body extract (0.5 g/kg, 1.0 g/kg, and 2.0 g/kg) and* Rhodiola rosea* (0.5 g/kg) were analyzed through rotating rod test (a), forced running test (b), and forced swimming test (c). Data are expressed as mean ± S.D. (*n* = 10) and analyzed using one-way ANOVA followed by Dunn's test. ^*∗*^ *P* < 0.05 and ^*∗∗*^ *P* < 0.01 versus nontreated mouse (Control).

Rotating rod, forced swimming, and forced running test were performed to detect the antifatigue capacity of CM. 0.5 mg/kg* Rhodiola rosea* which significantly enhanced the residence time in all three tests. Similar to* Rhodiola rosea*, compared with nontreated mice, 2.0 g/kg CM treatment improved nearly 100.2% and 115.8% residence time in female and male mice in rotating rod test (*P* < 0.05; Figure 1(a)). In forced running test, the running times were strongly enhanced after 2-week CM administration (*P* < 0.05; Figure 1(b)). In forced swimming test, CM treatment strongly enhanced swimming time with maximum record of 3.0 min and 3.4 min in female and male mouse compared with nontreated group (*P* < 0.01; Figure 1(c)).

### 3.2. The Antioxidant Effects of* Cordyceps militaris* before and after Swimming

Antioxidant enzymes including GSH-Px and SOD play important roles in preventing oxidative injury in animals [18, 19]. Overproduction of a large amount ROS leads to oxidative stress and deleterious effects on tissue [20]. To investigate the effect of CM on oxidative system, the content of MDA, activities of SOD and GSH-Px in serum, liver, and muscle, and the level of ROS in liver and muscle were determined. Two-week CM treatment enhanced serum GSH-Px activity in female and male mouse (*P* < 0.05; Table 1) and reduced serum MDA level in male mouse (*P* < 0.05; Table 1) before 20 min swimming. After swimming exercise, compared to controls, lower MDA level and higher SOD and GSH-Px activities were observed in CM-treated groups (*P* < 0.05; Table 1). However,* Rhodiola rosea* failed to influence the serum concentration of MDA and the activity of SOD in males (Table 1).

In liver, before swimming, 2.0 g/kg CM administration strongly reduced the level of ROS and enhanced the activity of GSH-Px in female and male mouse (*P* < 0.05; Table 2). The suppressive effect of CM on MDA level was only noted in female mouse (*P* < 0.05; Table 2).* Rhodiola rosea* displayed no effects on the activities of selected oxidant enzyme. After 20 min swimming, no differences of GSH-Px activity were found among all groups (*P* > 0.05). In contrast, 14 day CM treatment strikingly reduced the levels of MDA and ROS and enhanced the activity of SOD (*P* < 0.05; Table 2) which showed better antioxidant activity than that of* Rhodiola rosea*.

In muscle, similar as* Rhodiola rosea*, 2 g/kg CM administration enhanced GSH-Px activity in male mouse (*P* < 0.05; Table 3). However, after 20 min swimming, compared to nontreated group, lower MDA and ROS levels and higher SOD and GSH-Px activities were noted in CM-treated mouse (*P* < 0.05; Table 3) indicating CM displays strong antioxidant effect during relief fatigue.

### 3.3. The Regulation Effects of* Cordyceps militaris* on LD, LDH, and ATP

**Figure 2:**
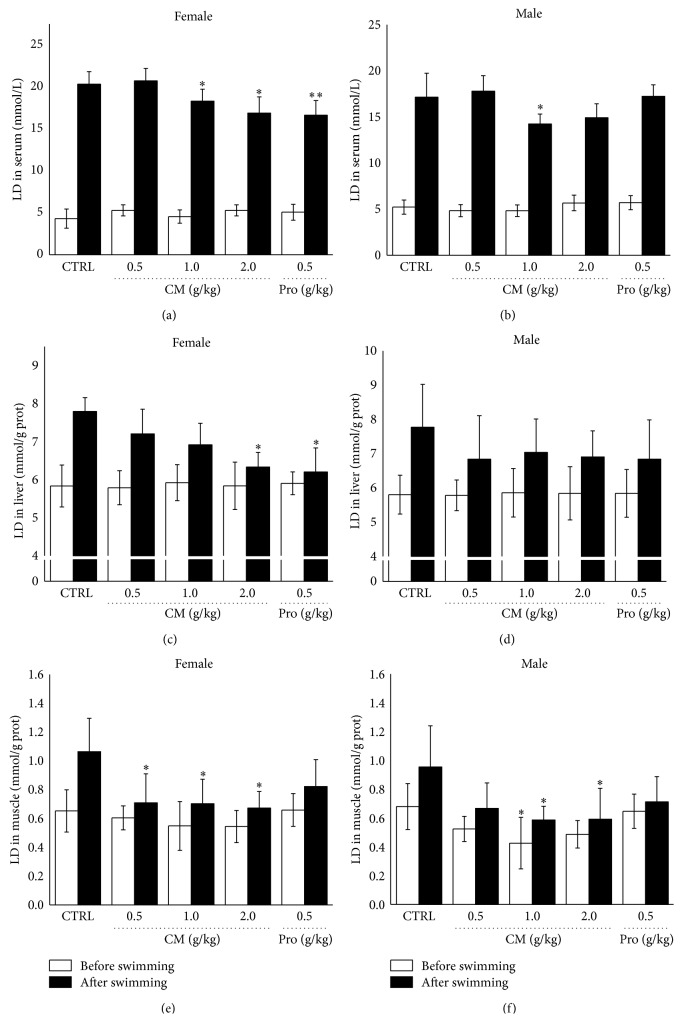
Treatment with* Cordyceps militaris* fruit body extract (0.5 g/kg, 1.0 g/kg, and 2.0 g/kg) and* Rhodiola rosea* (0.5 g/kg) for 2 weeks, before and after 20 min swimming, the levels of lactic acid in serum ((a), (b)), liver ((c), (d)), and muscle ((e), (f)) in male and female mouse were analyzed, respectively. Data are expressed as mean ± S.D. (*n* = 6) and analyzed using one-way ANOVA followed by Dunn's test. ^*∗*^ *P* < 0.05 and ^*∗∗*^ *P* < 0.01 versus nontreated mouse (Control).

The accumulation of lactate interferes with nerve impulses and muscle contraction, thus resulting in fatigue [21]. Before swimming, no significant differences on LD levels in serum, liver, and muscle were noted in female and male mice among all groups except for in muscle tissue of male mouse (Figure 2). After 20 min exercise, compared with nontreated mouse, 17.0%, 18.7%, and 36.6% reduction on LD level in serum, liver, and muscle were observed in 2.0 g/kg CM treated female mouse, respectively (*P* < 0.05; Figures 2(a), 2(b), and 2(c)). Interestingly, in male mouse, CM at dose of 1.0 g/kg only reduced 17.0% and 38.9% LD level in serum and muscle (*P* < 0.05; Figures 2(d) and 2(f)).* Rhodiola rosea* showed reductive effect on LD level only in serum and liver of female mouse (*P* < 0.05; Figures 2(a) and 2(b)).

**Figure 3:**
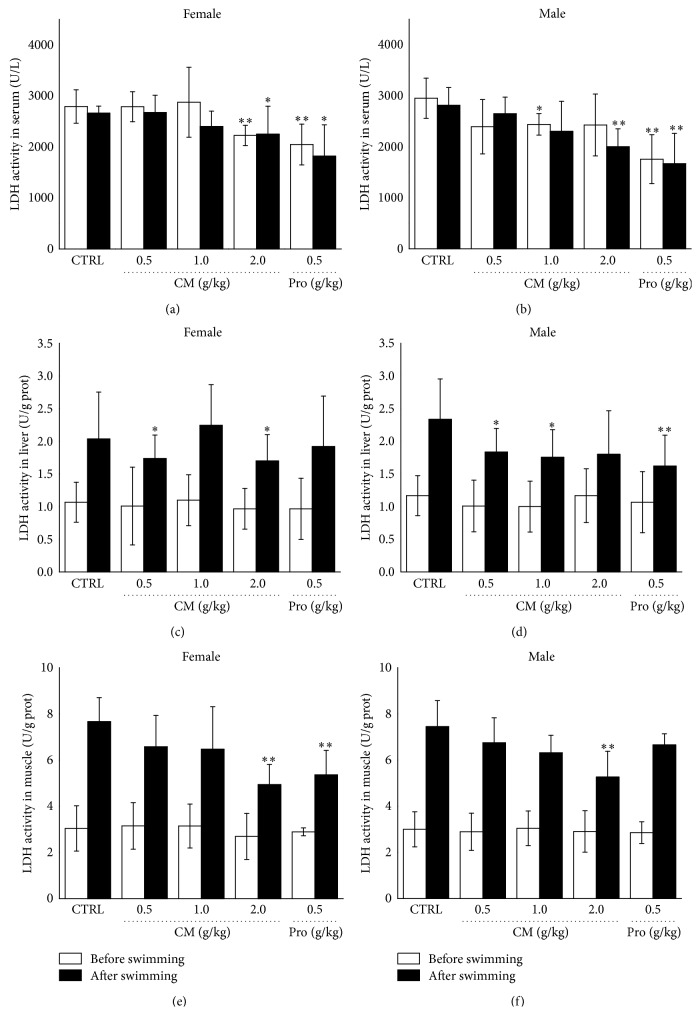
Treatment with* Cordyceps militaris* fruit body extract (0.5 g/kg, 1.0 g/kg, and 2.0 g/kg) and* Rhodiola rosea* (0.5 g/kg) for 2 weeks, before and after 20 min swimming, the levels of lactate dehydrogenase in serum ((a), (b)), liver ((c), (d)), and muscle ((e), (f)) in male and female mouse were analyzed, respectively. Data are expressed as mean ± S.D. (*n* = 6) and analyzed using one-way ANOVA followed by Dunn's test. ^*∗*^ *P* < 0.05 and ^*∗∗*^ *P* < 0.01 versus nontreated mouse (Control).

LDH, an accurate indicator of muscle damage, catalyzes the interconversion of pyruvate and lactate [22], which is determined in serum, liver, and muscle of all experimental mice. Before excise, compared to control group, 14-day CM treatment reduced 20.3% (at a dose of 2.0 mg/kg) and 17.4% (at a dose of 1.0 mg/kg) serum LDH activity in female mouse and male mouse, respectively (*P* < 0.05; Figures 3(a) and 3(d)). Similarly, 0.5 g/kg* Rhodiola rosea* only influenced serum LDH activity before swimming. After 20 min exercise, in CM-treated male and female mouse, LDH activity was suppressed in serum, liver, and muscle in different degree (*P* < 0.05; Figure 3).

**Figure 4:**
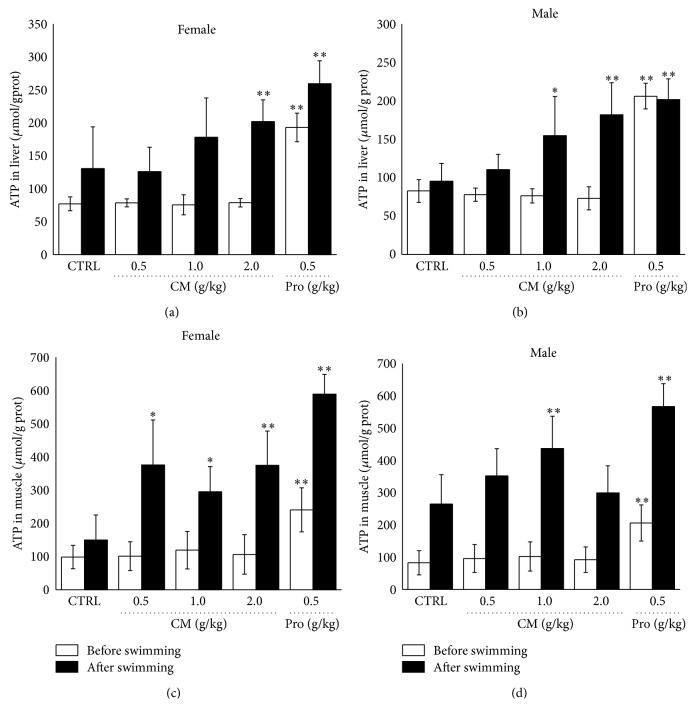
After 14-day* Cordyceps militaris* fruit body extract (0.5 g/kg, 1.0 g/kg, and 2.0 g/kg) and* Rhodiola rosea* (0.5 g/kg) treatment, the effects on ATP metabolism were analyzed in liver ((a), (b)) and muscle ((c), (d)) in both male and female mouse before and after 20 min swimming. Data are expressed as mean ± S.D. (*n* = 6) and analyzed using one-way ANOVA followed by Dunn's test. ^*∗*^ *P* < 0.05 and ^*∗∗*^ *P* < 0.01 versus nontreated mouse (Control).

ATP is the most direct and rapid energy source to exercise. The higher level of ATP protects the muscle against membrane damage, and the increase in LDH release is associated with a decrease in the content of ATP [23]. After swimming, 2.0 g/kg CM treatment enhanced approximate 54.6% and 151.2% ATP concentration in liver and muscle compared with control group in female mouse (*P* < 0.05; Figures 4(a) and 4(b)) and 1.0 g/kg CM treatment enhanced 90.8% and 65.2% ATP concentration in liver and muscle compared with nontreated male mouse (*P* < 0.05; Figures 4(c) and 4(d)).

### 3.4. The Regulation Effects of* Cordyceps militaris* on Protein Activations in Liver

**Figure 5:**
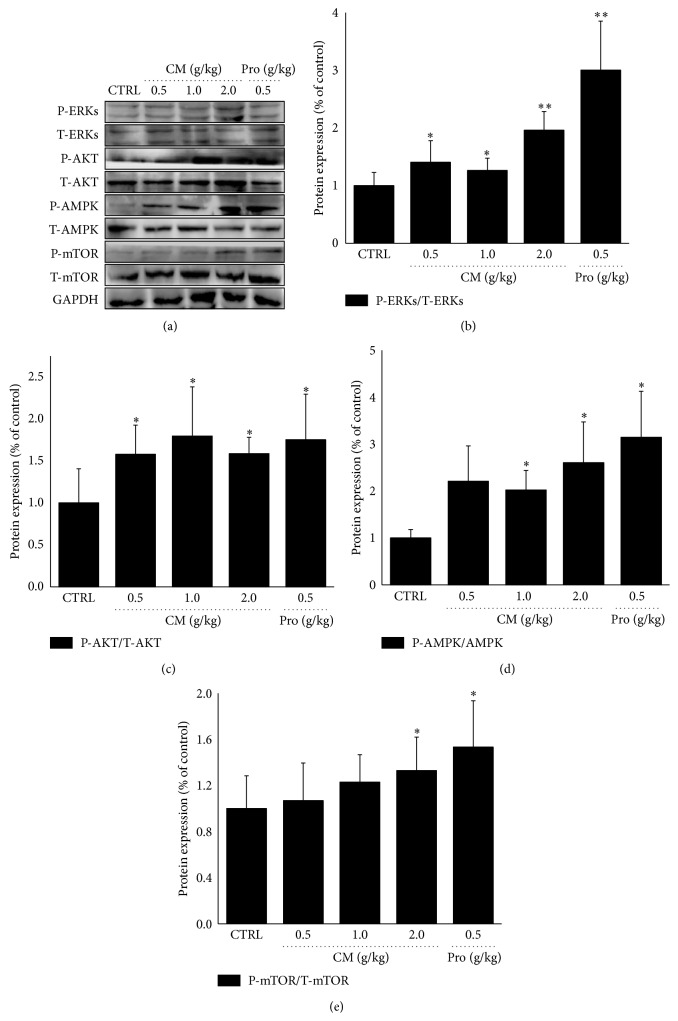
Mice were treated with* Cordyceps militaris* fruit body extract (0.5 g/kg, 1.0 g/kg, and 2.0 g/kg) and* Rhodiola rosea* (0.5 g/kg) for 14 days. After 20 min swimming, the activations of ERKs, AKT, mTOR, and AMPK in liver tissue were detected via western blot. Quantification data of the expression of P-ERKs, P-AKT, P-mTOR, and P-AMPK were normalized by corresponding T-ERKs, T-AKT, T-mTOR, and T-AMPK. Data are expressed as mean ± S.D. (*n* = 6) and analyzed using one-way ANOVA followed by Dunn's test. ^*∗*^ *P* < 0.05 and ^*∗∗*^ *P* < 0.01 versus control group.

In order to investigate the preliminary mechanisms during CM-mediated antifatigue activity, the phosphorylation of ERKs, AKT, mTOR, and AMPK in liver tissue was detected via western blot. Similar to the enhanced activity of* Rhodiola rosea* on protein levels, 14-day CM administration resulted in 96.2%, 79.6%, 32.9%, and 160.5% increment on the expressions of P-ERKs, P-AKT, P-mTOR, and P-AMPK (*P* < 0.05; Figure 5).

### 3.5. The Effects of* Cordyceps militaris* on Serum Hormones Concentration

**Figure 6:**
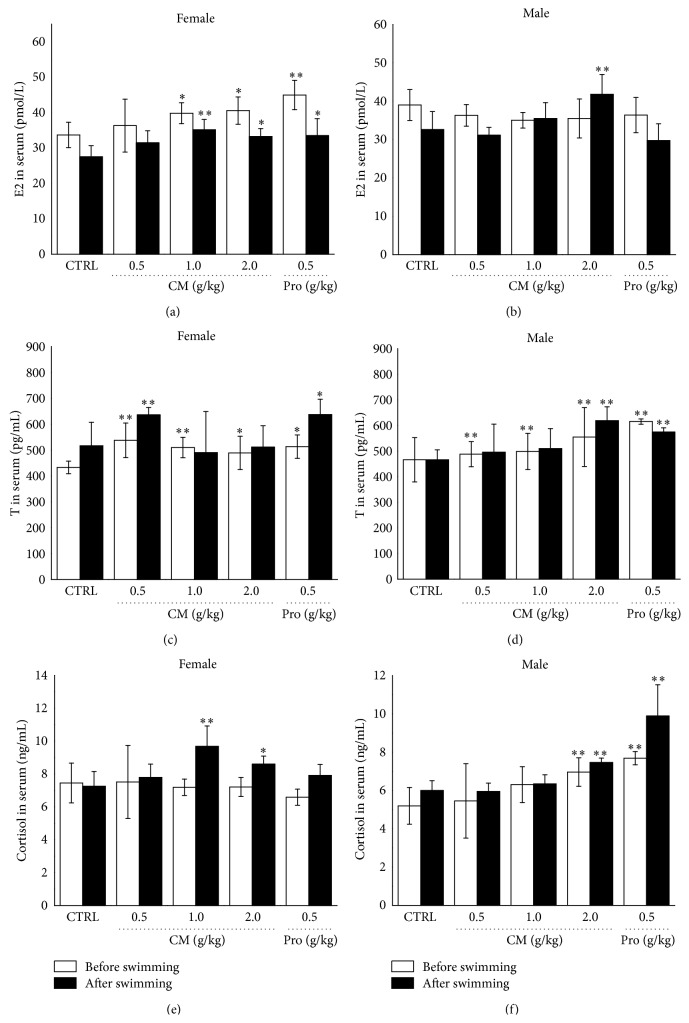
Mice were treated with* Cordyceps militaris* fruit body extract (0.5 g/kg, 1.0 g/kg, and 2.0 g/kg) and* Rhodiola rosea* (0.5 g/kg) for 14 days. Before and after 20 min swimming, the serum levels of estradiol ((a), (b)), testosterone ((c), (d)), and cortisol ((e), (f)) in both male and female mouse were determined via ELISA method. Data are expressed as mean ± S.D. (*n* = 6) and analyzed using one-way ANOVA followed by Dunn's test. ^*∗*^ *P* < 0.05 and ^*∗∗*^ *P* < 0.01 versus control group.

Since CM displayed different efficacy in male and female mouse, serum hormones including E2, T, and cortisol were detected after 14-day CM treatment. Both CM and* Rhodiola rosea* treatment enhanced serum E2 concentration in female mouse before and after 20 min swimming (*P* < 0.05; Figure 6(a)). Interestingly, compared to nontreated group, only CM at a dose of 2.0 g/kg increase 15.1% serum E2 in male mouse after swimming (*P* < 0.05; Figure 6(b)). In male and female mouse, the increased serum T levels were observed in CM and* Rhodiola rosea*-treated group before and after exercise (*P* < 0.05; Figures 6(c) and 6(d)). For serum cortisol level, treatment with 1.0 g/kg and 2.0 g/kg CM resulted in 33.5% and 18.6% increment after swimming in female mouse (*P* < 0.05; Figure 6(e)); however, in male mouse, 2.0 g/kg CM treatment increased nearly 34.6% and 24.5% before and after exercise (*P* < 0.05; Figure 6(f)).

## 4. Discussion

Overexercise and/or life pressure produced a large number of ROSs in human body which leads to irreversible tissue damage [24, 25]. Recently, herbs turn out to be a valuable reservoir for novel drugs selection to alleviate the symptoms of fatigue [26].* Cordyceps militaris* (L.) Link, a unique medicinal tonic fungus, possesses various pharmacological activities. Our separated experiment has successfully demonstrated that purified* Cordyceps militaris* polysaccharides showed antihypoxia effect in mouse [24]. Our present study aims to investigate the antifatigue activity of polysaccharides-enriched* Cordyceps militaris* water extract.

Through the metabolism, 2%-3% oxygen took into human body is translated into oxygen free radicals (OFR). On one hand, as signal transduction molecules, OFR participates in normal physiological process; on the other hand, superabundant of OFR causes oxidative stress [27], cell damage, and muscle fatigue. As reported, to maintain homeostasis, overaccumulated OFR can be cleared by antioxidant enzymes including SOD and GSH-Px. SOD catalyzes the conversion of superoxide into hydrogen peroxide and oxygen; meanwhile, GSH-Px scavenges the hydroxyl radical to maintain reduction-oxidation homeostasis [27, 28]. In our study, oral administration of* Cordyceps militaris* increased the activities of SOD and GSH-Px and decreased the levels of MDA and ROS in serum, liver, and muscle compared with nontreated mouse. Consistent with previous study,* Cordyceps sinensis* scavenges ROS, hydroxyl radicals, and superoxide anion via inhibiting MDA processing [29]. High intensity or exhaustive exercise is responsible for the hyperlevel of ROS in human body [20]. Clearing excessive MDA and ROS, SOD, and GSH-Px can contribute to the protection on cell oxidative damage. Oral administration of antioxidants has been demonstrated to reduce oxidative stress, relieve muscle fatigue, and improve exercise performance [8, 13]. Collectively, CM-mediated antifatigue effect may be associated with its antioxidant activity.

Our study revealed that CM at chosen doses regulated LD level and LDH activity in serum, liver, and muscle and enhanced ATP concentration in both liver and muscle. ATP is known as the most direct and rapid energy source to exercise. Acute and/or exhaustion exercise elevates muscle [H^+^] and depresses muscle function by inhibiting myofibrillar ATPase, which leads to the decrease of ATP synthesis [30]. LD, known as glycolysis product of carbohydrate under anaerobic conditions, is one of the major factors responsible for physical exercise-induced fatigue [22]. LDH which normally exists in muscle cell would be released into blood stream as a result of muscle damage [31]. In hematologic system, LDH oxidizes LD, changes the pH value, and further reduces LD-caused damage [32]. Therefore, LDH can be served as an indicator for fatigue determination [31]. Most antifatigue drugs display inhibitory effect on LD accumulation; similarly, CM that accelerated the clearance of LD may be involved in its alleviating fatigue phenomenon in experimental mouse.

In liver tissue, 14-day CM administration enhanced the phosphorylation of mTOR, AKT, ERKs, and AMPK after 20 min swimming. In starvation and/or oxidative stress conditions, the activated AMPK successfully promotes cell survival [33]. AMPK agonist AICAR is reported to regulate metabolic genes expression and improve exercise performance [34]. As a “cell energy regulator,” AMPK maintains ATP balance via inhibiting the synthesis of glycogen, cholesterol, and fat and promoting fatty acid oxidation and glucose transporter [7]. Once activated by abnormal cellular energy status, AMPK activates catabolic pathways to regulate ATP generation and consumption [35]. Previous research indicates that AMPK activation counteracts oxidative stress via suppressing ROS accumulation and increasing SOD and GSH-Px activities in liver [36]. Altogether, CM-mediated antioxidant and ATP enhancement is at least partially combined with AMPK phosphorylation which further influences its antifatigue effectiveness. Moreover, the enhancement of the expressions of P-AKT and P-mTOR was observed in our research. mTOR is considered as a downstream target protein of AKT, being sensitive to regulate growth factors and energy metabolism [37]. During exercise, AKT/mTOR signaling is activated to promote protein synthesis and translation [38]. Although our results suggest AKT/mTOR signaling is involved in the antifatigue activities of CM, we fail to explain the relationship between AMPK and AKT/mTOR. Moreover, the role of ERKs during the antifatigue activity still needs further investigation.

In our experiment, CM seems to be more efficiency in female mouse. We try to explain this phenomenon via analyzing serum hormone concentration. Exercise increases the hypothalamic-pituitary-testicular axis activity and promotes testosterone secretion. Consequently, testosterone improves exercise capacity, increases muscle strength, and delays fatigue occurrence [39]. Exogenous androgen has been confirmed to accelerate the recovery of fatigue [40]. Serum cortisol concentration is related to the form, intensity, and duration of exercise [41]. Our data demonstrate that the regulation of hormone level is involved in CM-mediated antifatigue effect. After CM treatment, different changing trends of E2 and cortisol concentration in serum were noted in male and female mouse which may explain the sex differences in response to CM-mediated antifatigue activities.

In conclusion, we have successfully confirmed that* Cordyceps militaris* induces fatigue recovery via activating AMPK and AKT/mTOR pathways and regulating serum hormone level. Our data provides experimental evidence in supporting clinical use of* Cordyceps militaris* as an effective agent against fatigue.

## Figures and Tables

**Table 1:** The effects of* Cordyceps militaris* fruit body extract on antioxidant status in serum of mice before and after swimming.

	Female					Male				
	CTRL	CM (g/kg)			Pro (g/kg)	CTRL	CM (g/kg)			Pro (g/kg)
		0.5	1.0	2.0	0.5		0.5	1.0	2.0	0.5
Before swimming										
MDA (nmol/mL)	17.36 ± 5.28	10.29 ± 3.25	12.41 ± 4.01	9.20 ± 4.55	12.90 ± 3.81	38.70 ± 4.04	20.5 ± 6.81^*∗∗*^	24.76 ± 9.52^*∗*^	9.52 ± 3.33^*∗∗*^	18.79 ± 4.91^*∗∗*^
SOD (U/mL)	96 ± 31	103 ± 13	99 ± 13	100 ± 33	126 ± 18^*∗∗*^	116 ± 20	113 ± 24	95 ± 20	89 ± 16	120 ± 23^*∗*^
GSH-Px (*μ*mol/mL)	81 ± 30	132 ± 32^*∗*^	156 ± 33^*∗∗*^	149 ± 33^*∗∗*^	150 ± 34^*∗*^	60 ± 5	65 ± 23	150 ± 36^*∗∗*^	139 ± 42^*∗*^	226 ± 34^*∗∗*^
After swimming										
MDA (nmol/mL)	36.19 ± 4.40	31.07 ± 5.98	11.62 ± 5.94^*∗∗*^	19.33 ± 4.74^*∗∗*^	37.78 ± 10.02	33.90 ± 4.29	32.38 ± 5.89	13.97 ± 4.86^*∗∗*^	21.67 ± 5.21^*∗∗*^	27.43 ± 3.94
SOD (U/mL)	76 ± 14	106 ± 21^*∗*^	127 ± 11^*∗∗*^	123 ± 20^*∗∗*^	110 ± 15^*∗*^	73 ± 21	140 ± 17^*∗∗*^	134 ± 9^*∗∗*^	102 ± 22^*∗*^	82 ± 17
GSH-Px (*μ*mol/mL)	132 ± 41	247 ± 42^*∗*^	280 ± 35^*∗∗*^	256 ± 59^*∗∗*^	350 ± 45^*∗∗*^	138 ± 45	269 ± 46^*∗∗*^	320 ± 60^*∗∗*^	295 ± 53^*∗∗*^	387 ± 45^*∗∗*^

Treatment with *Cordyceps militaris* fruit body extract (0.5 g/kg, 1.0 g/kg, and 2.0 g/kg) and *Rhodiola rosea* (0.5 g/kg) for 2 weeks; before and after 20 min swimming, the levels of MDA and the activities of SOD and GSH-Px in serum in male and female mouse were detected. Values are expressed as mean ± S.D. (*n* = 6). ^*∗*^ *P* < 0.05 and ^*∗∗*^ *P* < 0.01 compared with control group.

**Table 2:** The effects of *Cordyceps militaris* fruit body extract on liver antioxidant status in mice before and after swimming.

	Female					Male				
	CTRL	CM (g/kg)			Pro (g/kg)	CTRL	CM (g/kg)			Pro (g/kg)
		0.5	1.0	2.0	0.5		0.5	1.0	2.0	0.5
Before swimming										
MDA (nmol/mg prot)	0.53 ± 0.18	0.40 ± 0.08	0.25 ± 0.07^*∗*^	0.27 ± 0.15^*∗*^	0.50 ± 0.23	0.50 ± 0.17	0.39 ± 0.10	0.31 ± 0.12	0.30 ± 0.11	0.51 ± 0.09
ROS (FI/g prot)	1988 ± 203	1728 ± 278	1645 ± 158^*∗*^	1488 ± 202^*∗∗*^	1702 ± 189	1836 ± 147	1680 ± 203	1687 ± 256	1598 ± 108^*∗*^	1806 ± 217
SOD (U/mg prot)	196 ± 55	197 ± 16	196 ± 7	194 ± 17	201 ± 18	199 ± 16	194 ± 20	180 ± 15	174 ± 9	199 ± 15
GSH-Px (*μ*mol/g prot)	22 ± 7	24 ± 5	28 ± 7	29 ± 6^*∗*^	24 ± 5	24 ± 5	26 ± 4	29 ± 6	29 ± 3^*∗*^	26 ± 5
After swimming										
MDA (nmol/mg prot)	0.49 ± 0.075	0.36 ± 0.099	0.39 ± 0.104	0.21 ± 0.034^*∗∗*^	0.54 ± 10.02	0.76 ± 0.151	0.35 ± 0.089^*∗∗*^	0.43 ± 0.132^*∗*^	0.32 ± 0.082^*∗∗*^	0.50 ± 0.18
ROS (FI/g prot)	2128 ± 287	1928 ± 387	1351 ± 127^*∗∗*^	1288 ± 122^*∗∗*^	1601 ± 265^*∗*^	2158 ± 333	1590 ± 165^*∗∗*^	1580 ± 336^*∗*^	1317 ± 108^*∗∗*^	1606 ± 323^*∗*^
SOD (U/mg prot)	216 ± 73	196 ± 15	214 ± 84	323 ± 41^*∗*^	364 ± 94^*∗*^	146 ± 36	152 ± 47	231 ± 66^*∗*^	302 ± 35^*∗∗*^	349 ± 60^*∗∗*^
GSH-Px (*μ*mol/g prot)	37 ± 7	29 ± 7	31 ± 5	31 ± 10	46 ± 16	30 ± 5	34 ± 8	30 ± 3	30 ± 6	36 ± 8

Treatment with *Cordyceps militaris* fruit body extract (0.5 g/kg, 1.0 g/kg, and 2.0 g/kg) and *Rhodiola rosea* (0.5 g/kg) for 2 weeks; before and after 20 min swimming, the levels of MDA, ROS, and the activities of SOD and GSH-Px in liver in male and female mouse were detected. Values are expressed as mean ± S.D. (*n* = 6). ^*∗*^ *P* < 0.05 and ^*∗∗*^ *P* < 0.01 compared with control group.

**Table 3:** The effects of *Cordyceps militaris* fruit body extract on skeletal muscle antioxidant status in mice before and after swimming.

	Female					Male				
	CTRL	CM (g/kg)			Pro (g/kg)	CTRL	CM (g/kg)			Pro (g/kg)
		0.5	1.0	2.0	0.5		0.5	1.0	2.0	0.5
Before swimming										
MDA (nmol/mg prot)	0.78 ± 0.23	0.71 ± 0.19	0.77 ± 0.19	0.65 ± 0.20^*∗*^	0.73 ± 0.16	0.80 ± 0.25	0.75 ± 0.14	0.70 ± 0.19	0.69 ± 0.17	0.72 ± 0.16
ROS (FI/g prot)	15634 ± 1256	14569 ± 2012	15046 ± 2456	14569 ± 2178	14123 ± 1098	16023 ± 1891	15698 ± 2541	15036 ± 2013	15786 ± 1689	14563 ± 1048
SOD (U/mg prot)	113 ± 13	117 ± 19	1165 ± 13	115 ± 19	139 ± 30	117 ± 13	119 ± 27	117 ± 11	124 ± 11	157 ± 21^*∗*^
GSH-Px (*μ*mol/g prot)	10 ± 2	11 ± 3	13 ± 2	12 ± 2	11 ± 4	9 ± 2	11 ± 2	11 ± 3	13 ± 1^*∗*^	13 ± 1^*∗*^
After swimming										
MDA (nmol/mg prot)	1.13 ± 0.46	0.77 ± 0.30	0.76 ± 0.31	0.69 ± 0.25^*∗*^	0.72 ± 0.34	1.16 ± 0.45	1.07 ± 0.42	0.62 ± 0.18^*∗∗*^	0.89 ± 0.13	0.79 ± 0.17
ROS (FI/g prot)	22108 ± 2918	17812 ± 2954	16933 ± 3149	15381 ± 138^*∗*^	17885 ± 5084	23018 ± 1491	22846 ± 1496	18057 ± 2310^*∗*^	17038 ± 2119^*∗∗*^	17313 ± 3852
SOD (U/mg prot)	98 ± 10	100 ± 10	116 ± 22	128 ± 9^*∗∗*^	117 ± 5^*∗*^	101 ± 5	98 ± 14	100 ± 10	111 ± 8^*∗*^	120 ± 9^*∗∗*^
GSH-Px (*μ*mol/g prot)	9 ± 2	13 ± 1^*∗*^	11 ± 3	14 ± 3^*∗∗*^	11 ± 2	9 ± 1	10 ± 1	11 ± 2^*∗*^	10 ± 2	11 ± 2^*∗*^

Treatment with *Cordyceps militaris* fruit body extract (0.5 g/kg, 1.0 g/kg, and 2.0 g/kg) and *Rhodiola rosea* (0.5 g/kg) for 2 weeks; before and after 20 min swimming, the levels of MDA, ROS, and the activities of SOD and GSH-Px in skeletal muscle in male and female mouse were detected. Values are expressed as mean ± S.D. (*n* = 6). ^*∗*^ *P* < 0.05 and ^*∗∗*^ *P* < 0.01 compared with control group.

## References

[1] Davis J. M., Bailey S. P. (1997). Possible mechanisms of central nervous system fatigue during exercise. *Medicine and Science in Sports and Exercise*.

[2] Huang W. C., Lin C. I., Chiu C. C., Lin Y., Huang H., Huang C. (2014). Chicken essence improves exercise performance and ameliorates physical fatigue. *Nutrients*.

[3] Tanaka M., Baba Y., Kataoka Y. (2008). Effects of (-)-epigallocatechin gallate in liver of an animal model of combined (physical and mental) fatigue. *Nutrition*.

[4] Kim K. M., Yu K. W., Kang D. H., Koh J. H., Hong B. S., Suh H. J. (2001). Anti-stress and anti-fatigue effects of fermented rice bran. *Bioscience, Biotechnology, and Biochemistry*.

[5] Wu C. Y., Chen R., Wang X. S., Shen B., Yue W., Wu Q. (2013). Antioxidant and anti-fatigue activities of phenolic extract from the seed coat of *Euryale ferox* Salisb. and identification of three phenolic compounds by LC-ESI-MS/MS. *Molecules*.

[6] Nieman D. C., Pedersen B. K. (1999). Exercise and immune function. Recent developments. *Sports Medicine*.

[7] Grahame Hardie D., Ashford M. L. J. (2014). AMPK: regulating energy balance at the cellular and whole body levels. *Physiology*.

[8] Powers S. K., DeRuisseau K. C., Quindry J., Hamilton K. L. (2004). Dietary antioxidants and exercise. *Journal of Sports Sciences*.

[9] Powers S. K., Hamilton K. (1999). Antioxidants and exercise. *Clinics in Sports Medicine*.

[10] Dékány M., Nemeskéri V., Györe I., Harbula I., Malomsoki J., Pucsok J. (2006). Antioxidant status of interval-trained athletes in various sports. *International Journal of Sports Medicine*.

[11] Green D. R., Reed J. C. (1998). Mitochondria and apoptosis. *Science*.

[12] Bogdanis G. C., Stavrinou P., Fatouros I. G. (2013). Short-term high-intensity interval exercise training attenuates oxidative stress responses and improves antioxidant status in healthy humans. *Food and Chemical Toxicology*.

[13] Huang S.-C., Lee F.-T., Kuo T.-Y., Yang J.-H., Chien C.-T. (2009). Attenuation of long-term *Rhodiola rosea* supplementation on exhaustive swimming-evoked oxidative stress in the rat. *Chinese Journal of Physiology*.

[14] Das S. K., Masuda M., Sakurai A., Sakakibara M. (2010). Medicinal uses of the mushroom *Cordyceps militaris*: current state and prospects. *Fitoterapia*.

[15] Ng T. B., Wang H. X. (2005). Pharmacological actions of *Cordyceps*, a prized folk medicine. *Journal of Pharmacy and Pharmacology*.

[16] Dong Y., Jing T., Meng Q. (2014). Studies on the antidiabetic activities of *Cordyceps militaris* extract in diet-streptozotocin-induced diabetic Sprague-Dawley rats. *BioMed Research International*.

[17] Dong Y., Hu S., Liu C. (2015). Purification of polysaccharides from *Cordyceps militaris* and their anti-hypoxic effect. *Molecular Medicine Reports*.

[18] Barreto T. O., Cleto L. S., Gioda C. R. (2012). Swim training does not protect mice from skeletal muscle oxidative damage following a maximum exercise test. *European Journal of Applied Physiology*.

[19] Powers S. K. S., Sollanek K. J., Wiggs M. P., Demirel H. A., Smuder A. J. (2014). Exercise-induced improvements in myocardial antioxidant capacity: the antioxidant players and cardioprotection. *Free Radical Research*.

[20] Skenderi K. P., Tsironi M., Lazaropoulou C. (2008). Changes in free radical generation and antioxidant capacity during ultramarathon foot race. *European Journal of Clinical Investigation*.

[21] Maclaren D. P., Gibson H., Parry-Billings M., Edwards R. H. (1989). A review of metabolic and physiological factors in fatigue. *Exercise and Sport Sciences Reviews*.

[22] Kim H., Park S., Han D. S., Park T. (2003). Octacosanol supplementation increases running endurance time and improves biochemical parameters after exhaustion in trained rats. *Journal of Medicinal Food*.

[23] Fredsted A., Gissel H., Madsen K., Clausen T. (2007). Causes of excitation-induced muscle cell damage in isometric contractions: Mechanical stress or calcium overload?. *American Journal of Physiology—Regulatory Integrative and Comparative Physiology*.

[24] Davies K. J. A., Quintanilha A. T., Brooks G. A., Packer L. (1982). Free radicals and tissue damage produced by exercise. *Biochemical and Biophysical Research Communications*.

[25] Liu J., Yeo H. C., Övervik-Douki E. (2000). Chronically and acutely exercised rats: biomarkers of oxidative stress and endogenous antioxidants. *Journal of Applied Physiology*.

[26] Horng C., Huang J., Wang H., Huang C., Chen F. (2014). Antioxidant and antifatigue activities of *Polygonatum Alte-lobatum* hayata rhizomes in rats. *Nutrients*.

[27] Whaley-Connell A., McCullough P. A., Sowers J. R. (2011). The role of oxidative stress in the metabolic syndrome. *Reviews in Cardiovascular Medicine*.

[28] Manikandan S., Srikumar R., Parthasarathy N. J., Sheela Devi R. (2005). Protective effect of *Acorus calamus* Linn on free radical scavengers and lipid peroxidation in discrete regions of brain against noise stress exposed rat. *Biological and Pharmaceutical Bulletin*.

[29] Singh M., Tulsawani R., Koganti P., Chauhan A., Manickam M., Misra K. (2013). *Cordyceps sinensis* increases hypoxia tolerance by inducing heme oxygenase-1 and metallothionein via Nrf2 activation in human lung epithelial cells. *BioMed Research International*.

[30] Zhang L., Wan N. (2006). Advances in the research of sport fatigue caused by the action of free radical lipid oxidation. *Chinese Journal of Laboratory Diagnosis*.

[31] Choi E. H., Kang J. I., Cho J. Y. (2012). Supplementation of standardized lipid-soluble extract from maca (*Lepidium meyenii*) increases swimming endurance capacity in rats. *Journal of Functional Foods*.

[32] Huang L.-Z., Huang B.-K., Ye Q., Qin L.-P. (2011). Bioactivity-guided fractionation for anti-fatigue property of *Acanthopanax senticosus*. *Journal of Ethnopharmacology*.

[33] Wei Z., Peterson J. M., Wong G. W. (2011). Metabolic regulation by C1q/TNF-related protein-13 (CTRP13): activation of AMP-activated protein kinase and suppression of fatty acid-induced JNK signaling. *The Journal of Biological Chemistry*.

[34] McConell G. K., Manimmanakorn A., Lee-Young R. S., Kemp B. E., Linden K. C., Wadley G. D. (2008). Differential attenuation of AMPK activation during acute exercise following exercise training or AICAR treatment. *Journal of Applied Physiology*.

[35] Dërmaku-Sopjani M., Abazi S., Faggio C., Kolgeci J., Sopjani M. (2014). AMPK-sensitive cellular transport. *Journal of Biochemistry*.

[36] Bian Y.-F., Guo X.-X., Xiao C.-S. (2010). Protective effects of adiponectin against hypoxia/reoxygenation injury in neonatal rat cardiomyocytes. *Sheng Li Xue Bao*.

[37] Wullschleger S., Loewith R., Hall M. N. (2006). TOR signaling in growth and metabolism. *Cell*.

[38] Bolster D. R., Kubica N., Crozier S. J. (2003). Immediate response of mammalian target of rapamycin (mTOR)-mediated signalling following acute resistance exercise in rat skeletal muscle. *The Journal of Physiology*.

[39] Axell A.-M., MacLean H. E., Plant D. R. (2006). Continuous testosterone administration prevents skeletal muscle atrophy and enhances resistance to fatigue in orchidectomized male mice. *American Journal of Physiology—Endocrinology and Metabolism*.

[40] Cavallini G., Caracciolo S., Vitali G., Modenini F., Biagiotti G. (2004). Carnitine versus androgen administration in the treatment of sexual dysfunction, depressed mood, and fatigue associated with male aging. *Urology*.

[41] Kumari M., Badrick E., Chandola T. (2009). Cortisol secretion and fatigue: associations in a community based cohort. *Psychoneuroendocrinology*.

